# Focal Adhesion Kinase Intersects With the BRD4‐MYC Axis and YAP1 to Drive Tumor Cell Growth, Phenotypic Plasticity, Stemness, and Metastatic Potential in Colorectal Cancer

**DOI:** 10.1002/cam4.71227

**Published:** 2025-09-17

**Authors:** Rongbo Han, Junfeng Shi, Kai Cheng, Zian Wang, Yecang Chen, Orion Spellecy, Abu Saleh Mosa Faisal, Isha Aryal, Jinfei Chen, Rolf Craven, Olivier Thibault, Lauren Baldwin, Lawrence D. Brewer, Sonia Erfani, Chi Wang, Zhenheng Guo, Eric Chen, Burton Yang, Frederick Ueland, Ruihua Guo, Xiuwei Yang

**Affiliations:** ^1^ Department of Pharmacology and Nutritional Sciences College of Medicine, University of Kentucky Lexington Kentucky USA; ^2^ Department of Oncology, The Fourth Affiliated Hospital Nanjing Medical University Nanjing Jiangsu Province China; ^3^ Department of Oncology, Nanjing First Hospital Nanjing Medical University Nanjing Jiangsu Province China; ^4^ Department of Pathology, Nanjing Jinling Hospital Nanjing University School of Medicine Nanjing Jiangsu Province China; ^5^ College of Food Science and Technology Shanghai Ocean University Shanghai China; ^6^ Markey Cancer Center and Department of Toxicology and Cancer Biology, College of Medicine University of Kentucky Lexington Kentucky USA; ^7^ Department of Oncology The First Affiliated Hospital of Wenzhou Medical University Wenzhou Zhejiang China; ^8^ Pharmacy Services University of Kentucky Medical Center Lexington Kentucky USA; ^9^ Division of Medical Oncology and Hematology, Princess Margaret Cancer Center University of Toronto Toronto Ontario Canada; ^10^ Department of Laboratory Medicine and Pathobiology, Sunnybrook Research Institute University of Toronto Toronto Ontario Canada

**Keywords:** BRD4, colorectal cancer, FAK, metastasis, MYC, tumor growth

## Abstract

**Objective:**

Colorectal cancer (CRC) remains one of the leading causes of cancer‐related death worldwide due to the lack of effective therapies. Here we explored the clinical basis and therapeutic promise of the integrin‐focal adhesion kinase (FAK)‐dependent pathway for CRC.

**Methods and Results:**

Our bioinformatic and histological analyses showed that FAK was markedly upregulated at both mRNA and protein and signaling levels in the two CRC patient cohorts. Particularly, the portion of carcinomas carrying active FAK (Y^397^phosphorylation) increased by threefold from stage I to III/IV tumors or metastatic lesions. Consistent with this clinic landscape, FAK inhibition via knockdown or chemical inhibitors suppressed tumor cell growth largely in the subset of CRC cell lines with low MYC expression. In contrast, the FAK inhibition was less effective in the cell line pool with high MYC expression. The resistance to FAK targeting diminished upon a co‐inhibition of BRD4 via BET inhibitors. It coincided with an induction of cell cycle arrest at G1‐S and G2‐M phases, elevated apoptosis and chemosensitivity (paclitaxel and oxaliplatin), and impaired stemness. Mechanistically, the BET inhibitor induced an EMT‐like phenotype, tilting tumor cell dependence toward the integrin‐FAK axis. Moreover, inhibiting FAK alone or in combination with SRC or BRD4 markedly suppressed cell motility and the YAP or MYC activation, and restored the expression of the long isoform BRD4. Also, co‐genomic/genetic dysregulations of FAK and YAP1 or SRC strongly correlated with poor disease‐free patient survival.

**Conclusion:**

Overall, our study highlights the potent pro‐malignant role of the integrin‐FAK axis in CRC, fueling its targeting as a single agent or synthetic lethal‐based therapy.

## Graphical abstract

Functional and signaling roles of integrin‐FAK‐dependent pathway in colorectal cancer.

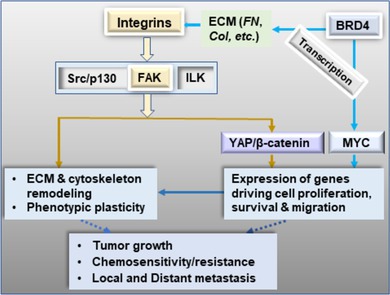

## 1 Introduction

Colorectal cancer (CRC), one of the most commonly diagnosed cancers globally, is the second leading cause of cancer‐related deaths [1]. This malignant disease is characterized by high genomic instability, which gives rise to a vast amount of genetic and epigenetic alterations and reprogramming of diverse biochemical and signaling networks [2, 3, 4]. Such a chaotic molecular landscape is strongly linked to clinical resistance to currently available therapies, including cytotoxic chemotherapy, antiangiogenic, and anti‐epidermal growth factor receptor (anti‐EGFR) therapies [2, 5]. Thus, there is an urgent need for an in‐depth mechanistic understanding of the disease and the development of novel therapies.

Integrins and associated pathways have been broadly investigated for their role across different stages of tumor development and progression in CRC and are increasingly regarded as promising therapeutic targets [6]. By engaging with extracellular matrix (ECM) ligands or neighboring cells, these heterodimeric adhesion receptors recruit Talin/Kindlin/ILK‐based protein complexes to drive cell adhesion and remodeling of the actin cytoskeleton, and to support tissue architecture and stiffness [7]. Physiologically, a number of integrins are shown to mediate the architecture of intestinal mucosa layers and their regeneration through sustaining the stem cell population [8, 9, 10, 11]. In CRC, the expression of some members of the family, such as β3 integrin, appears markedly dysregulated and is regarded as a hallmark for consensus molecular subtype 2 (CMS2), the most common form of the disease [6]. Other members of the integrin family, notably the β1 integrin‐based receptors, are also implicated in the regulation of colon cancer development and drug response [12, 13]. Functionally, integrins have been linked to the regulation of pro‐metastatic processes in CRC, including cell migration and invasion [13, 14, 15, 16, 17, 18]. Several members of the integrin family are implicated in the regulation of disease dormancy and reactivation of cancer stem cells in CRC [8, 12, 13, 19]. Thus, there is a large body of cellular and functional evidence implicating a critical role of integrins in tumorigenesis, metastatic progression, drug resistance, and disease recurrence in CRC.

Mechanistically, integrins have been probed regarding their impact on CRC development and metastasis and therapeutic potential at the signaling level. Notably, as one of the key signaling effectors of integrins, focal adhesion kinase (FAK) has been shown to drive tumor onset and growth in the adenomatous polyposis coli protein (APC)‐null mouse model [9, 20]. A similar role has been described for c‐Src, another key effector of integrin‐dependent signaling [10, 21]. Furthermore, both FAK and c‐Src are implicated in the promotion of drug resistance or disease progression in CRC [22, 23, 24]. The integrin‐FAK axis appears to mediate destabilization of β‐catenin‐based protein complexes in the cytosol and subsequent nuclear translocation, which in turn impacts the expression of arrays of pro‐tumorigenic genes, including cyclin D1 and MYC [9, 20]. It is also implicated in the regulation of tumor progression in CRC through the E‐cadherin/β‐catenin‐mediated adherent junction or cell–cell contact, and the epithelial‐mesenchymal transition (EMT) via the TGF‐β pathway or transcription factors such as Slug and Snail [5, 25]. Conceptually, the functional importance of integrin‐dependent pathways in CRC malignancy may be underestimated, as a high frequency of genetic mutations of E‐cadherin may shift tumor cell dependence on integrin‐mediated cell‐extracellular matrix (ECM) interactions for survival and therapeutic resistance [26]. Conceivably, the integrin‐FAK axis is a potential driver of a wide spectrum of pro‐tumorigenic and pro‐metastatic activities or processes in CRC and is worthy of further study of its molecular action and therapeutic potential.

In the current study, we explored pathological alterations, molecular roles, and therapeutic potential of the integrin‐FAK axis in CRC in the context of oncogenic activation of MYC and YAP1. The clinical link of the integrin‐FAK axis was probed by conducting bioinformatic and histological analyses of both The Cancer Genome Atlas (TCGA) and local CRC patient cohorts with respect to their genomic dysregulations, subcellular distribution/activation across tumor stages and metastatic lesions. The potential downstream effectors and therapeutic targeting of the integrin‐FAK axis in CRC were assessed by analyses of functional and signaling effects of gene knockdown and pharmacological inhibitors alone or in combinations with chemotherapeutic agents across a panel of representative cell line models. Data from our analyses unveil that the integrin‐FAK axis is frequently dysregulated in the human CRC population at genomic, genetic, and molecular levels, and these dysregulations correlate with tumor onset or disease progression. Importantly, the critical role of the axis in tumor cell proliferation, cell cycle progression, and survival, phenotypic plasticity, and chemoresistance is intrinsically tied to activation of YAP1 and the BRD4‐MYC axis. Altogether, our results illustrate a strong pro‐malignant role of the integrin‐FAK axis in CRC and provide crucial pathological and biological rationale for FAK/MYC‐based co‐targeting as a novel therapeutic strategy.

## 2 Materials and Methods

### 2.1 Cell Culture and Reagents

A panel of authenticated human colon cancer cell lines, including RKO, Caco2, DLD‐1, HCT116, LoVo, LS117T, HT‐29, SW480, and SW620, was obtained from ATCC (Manassas, VA).

MC38, a mouse CRC cell line, was purchased from Sigma‐Aldrich (St. Louis, MO, USA). All cell lines were cultured in RPMI 1604 or DMEM (Invitrogen) supplemented with 5%–10% FBS (Sigma‐Aldrich, St. Louis, MO, USA) under 37°C and 5% CO_2_. Stem culture medium was purchased from Lonza (Basel, Switzerland) for analysis of tumorsphere formation. Cell lines were periodically examined for Mycoplasma contamination by PCR analysis.

The sources and dilution of antibodies used in immunostaining were listed in Table S1. The antibodies used for western blotting, including those for detection of total or phosphorylated forms of FAK, YAP1, AKT, Bcl‐xl, β‐catenin, BRD4, and phosphorylated or ɣ‐H2Ax, were purchased from Cell Signaling Technology (Danvers, MA). The sources of Myc and E‐cadherin antibodies were described in a prior study [27]. Chemical inhibitors, including VS‐606, ABBV‐774, MK2206, GDC0941, dasatinib, and lapatinib, were obtained from MedChemExpress (Monmouth Junction, NJ). Chemotherapeutic agents, including 5‐FU, Oxaliplatin, Carboplatin, Paclitaxel, and Irinotecan, were purchased from Sigma‐Aldrich (St. Louis, MO). Additional small molecule inhibitors and antibodies used in the current study were purchased from the vendors listed in a prior study [26, 27].

### 2.2 The shRNA‐Mediated Gene Knockdown

FAK knockdown was carried out via the use of shRNAs obtained from Dharmacon (Boulder, Denver, CO, USA). Stable cell transfection was conducted using Lipofectamine 2000 (ThermoFisher, Waltham, MA, USA) and selected under 1–4 μg/mL puromycin.

### 2.3 Assays of Cell Viability, Cell Cycle, Apoptosis, Cell Motility, and Tumorsphere Formation

Effect of gene knockdown or chemical inhibitors on *cell viability* was performed according to the protocol described in a recent study [26]. In brief, tumor cells were seeded into a 96‐well plate overnight and subsequently treated with indicated doses of inhibitor. After 48–96 h, cell viability was assessed via the MTT assay [27]. For *cell cycle* analysis, cells were starved overnight, followed by inhibitor treatment for 36 h in the presence of 10% FBS. This was followed by subsequently staining with propidium iodide (10 μg/mL) and analysis on flow cytometry. To assess the extent of *apoptosis*, tumor cells were treated with DMSO or inhibitors for 48–72 h in growth medium, and stained with propidium iodide and APC‐conjugated Annexin V (10 μg/mL, BioLegend), and analyzed by flow cytometry. The inhibitor effect on cell motility was assessed by performing the wound healing assay. After tumor cells were grown to confluence in 24‐well plates, artificial wounds were generated and treated with inhibitors for 24–36 h prior to imaging microscopically.

The inhibitor effects on *tumorsphere formation* were assessed according to the protocol described in our prior study [28]. In brief, tumor cells were detached in trypsin–EDTA and neutralized with serum. After washing 3× in PBS, 1.0 × 10^3^ tumor cells were seeded into a single well of 24‐well ultralow adhesion plates overnight and cultured in stem cell medium as described in a prior study [29]. It was followed by treatment with chemical inhibitors for 2–5 days and imaging on a NIKON Eclipse microscope workstation.

### 2.4 Immunoblotting Analysis of Cell Signaling and Protein Expression

To assess changes in cell signaling, tumor cells were treated with chemical inhibitors or RNA oligos for the indicated time periods, followed by lysing in RIPA buffer in the presence of protease inhibitors and Na_3_VO_4_ [27]. Immunoblotting was performed by incubating lysates with primary and secondary antibodies, followed by detection using a chemiluminescence kit (Thermo‐Fisher). β‐actin was blotted as a sample loading control. Differences in protein level between groups or treatments were estimated via use of Image J software.

### 2.5 Bioinformatic Analysis of TCGA Dataset

Genomic and transcriptional alterations of interested genes, including differences in gene copy number and levels of mRNA expression, were probed by performing an analysis of the colon cancer patient cohort at the TCGA database (PanCancer Atlas) with the c‐Bio Portal platform [30].

### 2.6 Biospecimen Collection and IHC Analysis

The use of deidentified patients' biospecimens in the current study was granted exemption and approved by the institutional IRB ethics committee (SFY20220510‐K147). The biospecimens (paraffin‐embedded normal and malignant tumor tissues or the pleura fluids/ascites) and related clinical information described in the current study were collected with the informed consent of the patients diagnosed and/or treated in Nanjing Jinling Hospital/Nanjing University School of Medicine or Nanjing First/Forth Hospital/Nanjing Medical University. For ascites samples, the pleura fluids/ascites collected from patients were subjected to centrifugation for 5 min at 2000 rpm. The resulting pellets were immediately fixed in formalin and embedded in paraffin prior to IHC analysis. The patient sources of all the biospecimens remained deidentified or anonymous throughout the study. The details of the IHC analysis of these biospecimens were described in our prior studies [26]. All values used in the evaluation of differences in antibody staining between groups or stages were obtained by multiplying the percentage of positively stained tumor cells with score values of staining intensity. The criteria for estimating the percentage of immunoreactive cells were set as follows: 0, < 1%; 1, 1%–10%; 2, 11%–50%; 3, 51%–100%. In the study, the antibody staining intensity was scored as: 0, negative; 1, weak; 2, moderate; 3, strong.

### 2.7 Statistical Analyses

Kaplan–Meier curves and Log‐rank tests, along with Bonferroni correction, were employed to compare differences in the duration of patient survival between groups for the analysis of the TCGA cohort. ANOVA and multiple comparison analysis were performed for the evaluation of the difference in cell phase distribution between control and treatment groups. The *χ* ^2^ analysis was adopted to evaluate the degree of co‐expression of FAK, BRD4, MYC, β‐catenin, E‐cadherin, and YAP1 in tumor tissues from the local patient cohort.

## 3 Results

### 3.1 Genomic and Molecular Dysregulation of Integrins and Associated Pathways

**FIGURE 1:**
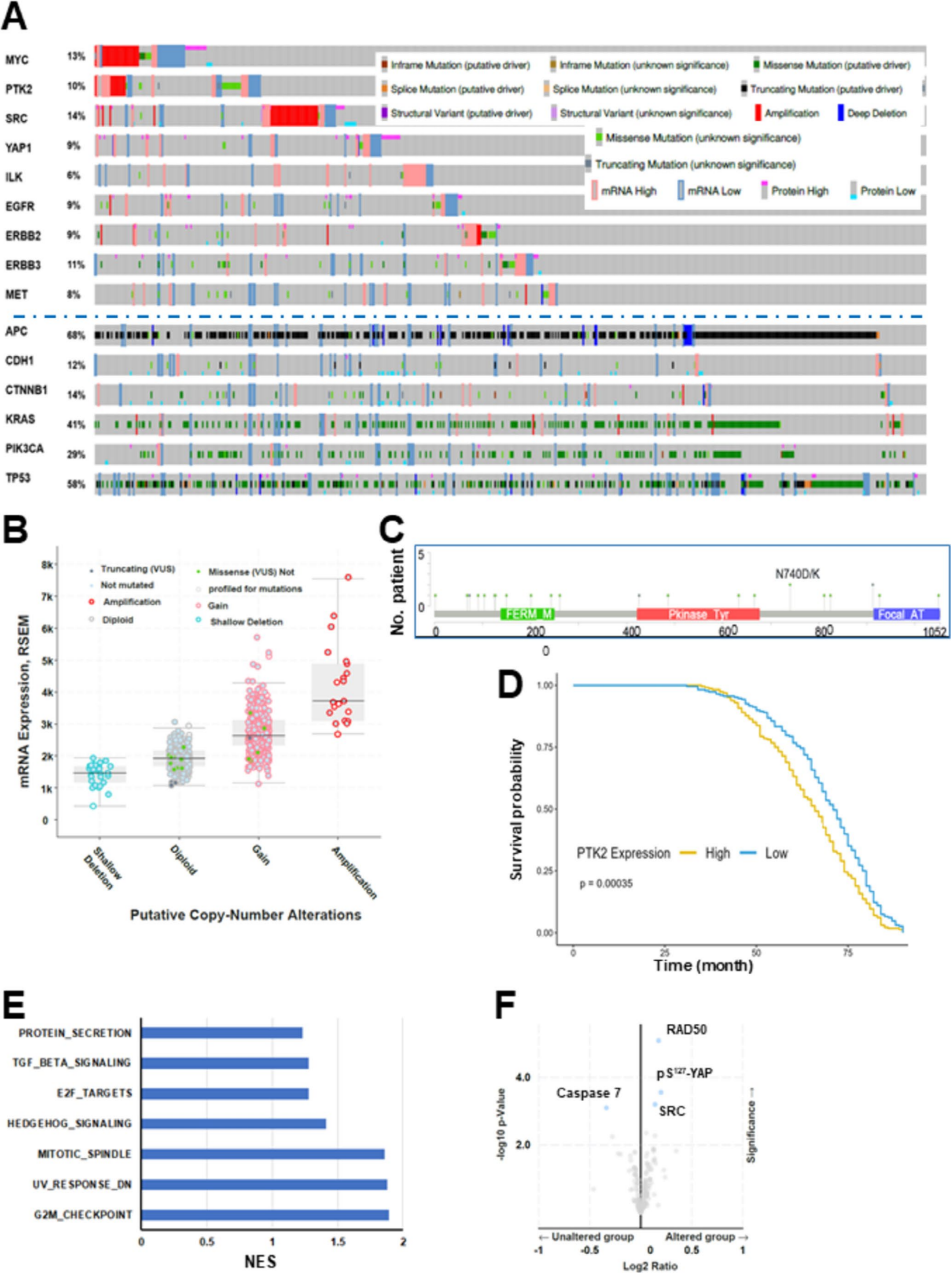
Probing genomic and transcriptional dysregulation of integrin‐dependent pathways and associated oncogenic pathways in a colorectal cancer patient cohort from the TCGA database. (A) Tumor samples with mutations and CNA data (*N* = 594 samples/patients) from a colorectal adenocarcinoma cohort (TCGA, PanCancer Atlas), which spanned all stages of CRC primary tumors (*N* = 594), were probed for altered level of mRNA, gene mutations, structural variants of FAK (PTK2) and other integrin‐dependent signaling effectors as well as oncogenic drivers via use of c‐Bioportal software. The threshold of *z*‐score relative to normal samples (log RNA Seq V2 RSEM) was set at ±2. (B) Association between the gene copy alterations of PTK2 (FAK) and mRNA expression in the cohort. (C) Occurrence of point mutations in FAK gene in the cohort. (D–F) The overall patient survival was plotted for the populations with high or low mRNA expression of FAK in the TCGA cohort. *Y axis*: Probability of patient survival. X axis: Duration of patient survival (months). Patient survival curves were compared using log‐rank test to assess differences in disease free survival (DFS) between the high vs. low expression groups. Hazard ratios (HR) with 95% CI were calculated to quantify the risk associated with high vs. low expression of relevant genes. The data was normalized by transcripts per million (TPM). A list of pathways and genes from the gene enrichment analysis via the Hallmark‐based logarithm (E, F). (F) X axis: Values of NES (FDR value < 0.05).

Dysregulation of integrins and associated FAK‐dependent signaling has been implicated in tumor development and metastatic disease progression in CRC [9, 20]. Yet, the underlying genetic basis and therapeutic relevance remain largely unclear. To address this void, we probed the genomic status (i.e., gene copy number alteration), mRNA expression, and related clinical outcomes for major effectors of the integrin‐dependent signaling pathway in the CRC patient cohort in the TCGA dataset. As shown in Figure 1A, 10%–14% of primary tumor samples exhibited copy number gain and aberrant mRNA expression in both FAK (PTK2) and SRC, consistent with prior studies [3, 4]. Of potential therapeutic importance, the amplification of MYC and FAK genes was partially overlapping, consistent with their adjacent localization on chromatin 8q24 arm. By comparison, such genomic alteration rarely occurred in YAP1 and ILK, two additional effectors of integrin‐mediated signaling [7]. SRC and YAP1 were also upregulated at the protein level in a significant portion of tumor samples in the cohort. The genomic changes in FAK/MYC and SRC or YAP1 rarely overlapped in the population.

The broad genomic alterations of integrin‐dependent pathways imply their clinical importance in CRC development and progression. Thus, we further evaluated the dynamics of the integrin‐FAK axis in the CRC cohort. As shown in Figure 1B, there was a strong association between the mRNA expression of FAK and its gene copy number alteration in the TCGA cohort. Even though various point mutations of FAK occurred across the open reading region, they appeared to have limited negative impact on its expression or activation, based on recent biochemical studies (Figure 1C) [31, 32]. Furthermore, we probed the clinical significance of aberrant expression of FAK by stratifying the CRC cohort into two groups: low and high mRNA expression of FAK in the TCGA cohort. As shown in Figure 1D, the patient group with high FAK expression (FAK^high^) exhibited significantly worse survival than its counterpart (FAK^low^) (*p* < 0.00035) Our gene enrichment analysis showed that the group with elevated FAK expression was accompanied by upregulation/activation of a group of genes involved in cell cycle progression through G1/S and G2/M, particularly transcription factors E2F, a family of well‐recognized targets of MYC oncogene (Figure 1E). There was also elevated expression of RAD50 and SRC or YAP activation (pS^127^) (Figure 1F). These data suggest a potential pro‐tumorigenic role of FAK in CRC.

**FIGURE 2:**
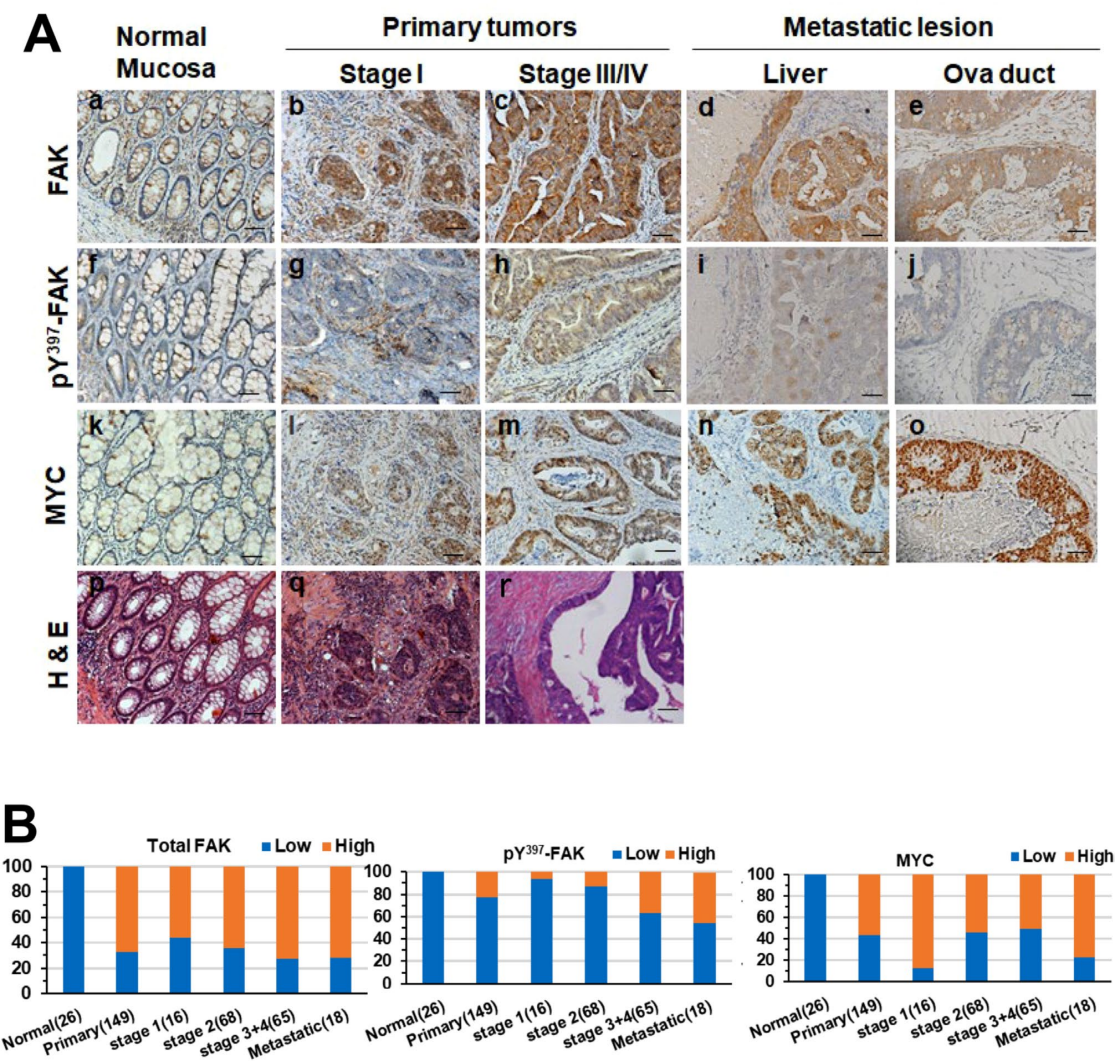
IHC analysis of total and active FAK and MYC expression in adenocarcinomas and distant metastatic lesions from a local CRC patient cohort. (A) Representative images of H&E and IHC staining for FAK, active FAK (pY397), and MYC in normal colonic mucosa or tumor‐adjacent tissues (a, f, k and p), stage I (b, g, I and q) and stage III/IV (c, h, m and r) colorectal adenocarcinomas, as well as liver (d, I and n) and oviduct (e, j and o) metastatic lesions. Panels show H&E (p, q, r), total FAK (a–e), pY397‐FAK (f–j), and MYC (k–o) staining. (B) Proportion of tumors with low (blue) or high (red) protein expression of total FAK, pY397‐FAK, and MYC across tumor stages in the CRC patient cohort (%). The x‐axis indicates the source of tissues and number of tumors examined. Antibody staining values are shown separately for cytoplasmic and nuclear expression of FAK, pY397‐FAK, and MYC. Scale bar: 100 μm.

To gain an in‐depth pathological insight into the dysregulation of the integrin‐FAK axis in CRC, we performed histological analysis to evaluate its expression and activation in another patient cohort. As shown in Figure 2A, the immunostaining of active or total FAK was exclusively localized in the cytosol of tumor cells. The level of total or active FAK was markedly elevated in a large portion of tumors (56%–72.3%) and metastatic lesions in our local CRC patient cohort, compared to adjacent normal intestinal mucosa. More strikingly, the percentage of tumors with active FAK (pY^397^ form) increased with tumor stage by nearly threefold (13% and 37.2%) (Figure 2A,B). These observations, combined with the output from the above genomic or histological analyses of CRC patient cohorts, further support a pro‐tumorigenic role of FAK and are consistent with a prior study of FAK using an in vivo animal mouse model [9].

Moreover, the subset of integrins driving cell‐ECM interactions, including α1/α2, α5/α11, αv and α6 which interact with collagen, fibronectin, vitronectin, and laminins, respectively, exhibited a marked upregulation at the mRNA level in primary tumors (Figure S1A). However, there were opposite changes in α3 and α4 or β2 integrins, which are known to mediate both cell‐ECM and cell–cell interactions or adhesion of hematopoietic cells. This data further supports the notion of the aberrant expression of β3 integrin as a hallmark of CMS subtype in CRC.

### 3.2 Probing the CRC Dependence on the Integrin‐FAK Axis

**FIGURE 3:**
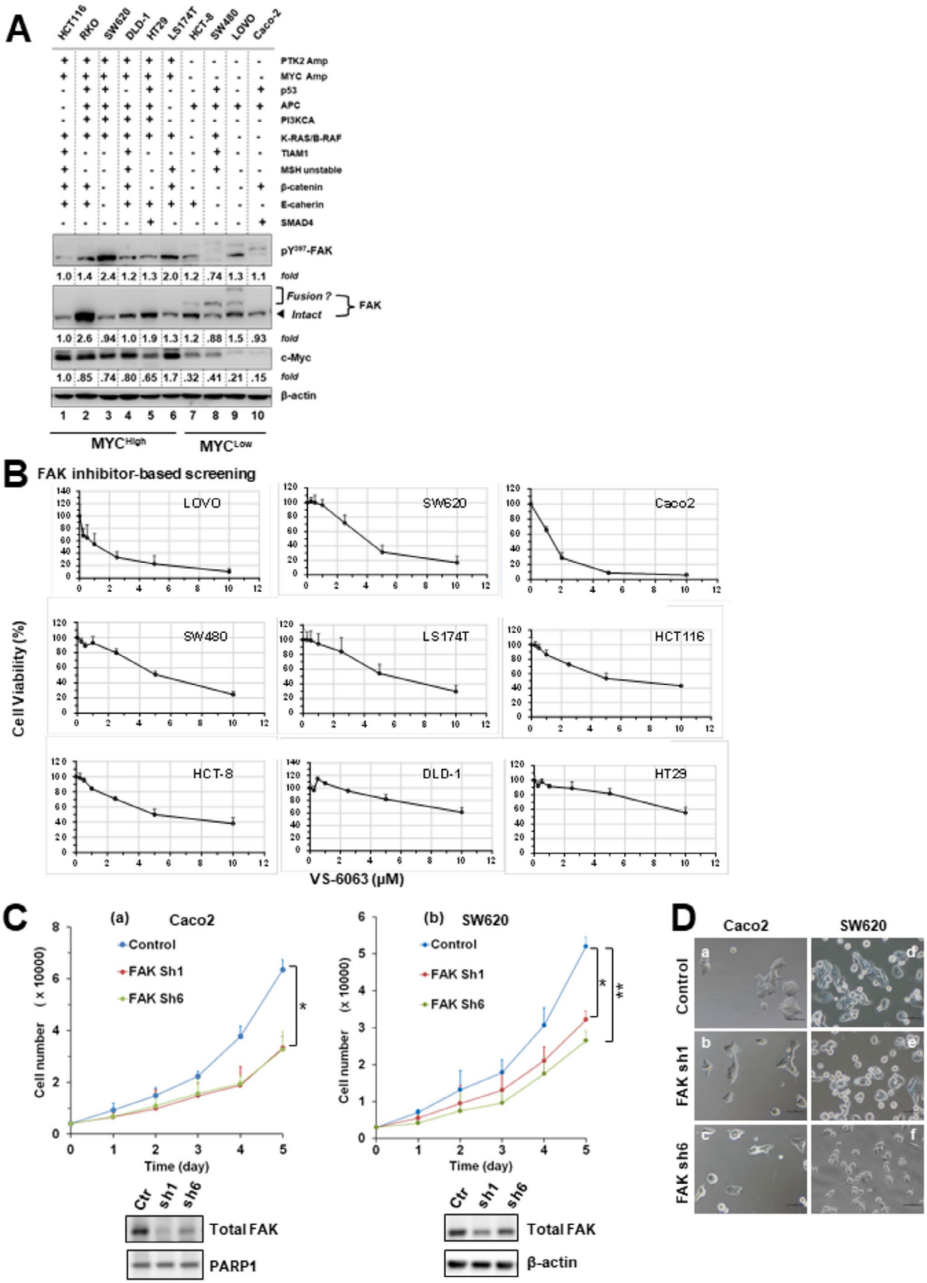
FAK dependence in colorectal adenocarcinoma cells. (A) Aberrant expression of total FAK, active FAK (pY397), and MYC across a panel of CRC cell lines analyzed by immunoblotting. Tumor cells were cultured in 6‐well plates, lysed, and probed with the indicated antibodies. Protein expression differences between cell lines were quantified by densitometry (OD values) relative to HCT116 (lane 1), normalized to β‐actin loading. Status of oncogenic drivers is indicated: *Amp*, gene amplification; additional oncogenes harboring point mutations or deletions are listed. Putative *PTK2–TRAPPC9* fusion gene products are also noted. (B) CRC cell line responses to escalating doses of the FAK inhibitor VS‐6063. Cells seeded in 48‐ or 96‐well plates were pre‐cultured overnight in 10% FBS and treated for 72 h with the indicated doses of VS‐6063. Viability was determined by MTT assay. Y‐axis: cell viability (% of control, 0.1% DMSO); mean ± SEM, *n* = 3. (C) Effect of FAK knockdown on proliferation. Caco2 and SW620 cells with (w) or without (w/o) stable FAK knockdown were seeded in 96‐well plates with 10% FBS and monitored for changes in cell number over time. Y‐axis: cell number; mean ± SEM, *n* = 3. Validation of FAK knockdown was confirmed by immunoblotting; β‐actin served as a loading control. Control: parental cells or cells treated with scramble shRNA. (D) Representative images of Caco2 (a–c) and SW620 (d–f) cells with or without stable FAK knockdown. Cells were seeded in 12‐well plates, cultured overnight, and imaged microscopically. *p* < 0.05; *p* < 0.01.

The FAK dependence or addiction of CRC tumors is implicated by our above bioinformatic and pathological analyses (Figures 1 and 2) and a prior genetic study with a mouse model featuring APC loss [9]. Whether this notion holds across a broad oncogenic context, particularly regarding the BRD4‐MYC axis, remains unknown. To address this question, we probed the status of total and active FAK (pY^397^‐FAK) and MYC across a panel of 10 human CRC cell lines by immunoblotting. As shown in Figure 3A, the expression of total FAK was particularly higher in cell lines carrying MYC amplification, such as RKO and HT‐29, based on the fold differences calculated via the densitometry analysis. The ratio of pY^397^‐FAK over total FAK level was highest in SW620, LOVO, and LS174T. In contrast, these CRC cell lines readily fell into two groups according to MYC expression: MYC^high^ and MYC^low^ groups. Interestingly, the IC_50_ values of VS‐6063, a pharmacological inhibitor of active FAK, were significantly lower in Caco2, LOVO, and SW620 lines among these cell lines examined (Figure 3B). These VS‐6063‐sensitive lines also expressed a low level of MYC protein and were highly responsive to FAK knockdown (Figure 3C), which led to a 40%–70% decrease in total FAK by immunoblotting analysis (Figure 3C). In contrast, such inhibitory effect was less apparent in the two MYC^high^ cell lines, DLD‐1 and HT‐29, even though they possessed a high level of active FAK (data not shown). Additionally, there were relatively high percentages of roundup cells in the FAK KD group than the control in Caco2 (b‐c) or SW620 (e‐f) lines (Figure 3D). Collectively, these data suggest that the FAK dependence occurs in a fraction of the MYC^low^ tumors in CRC.

### 3.3 Functional Cooperation Between FAK, MYC, and BRD4

**FIGURE 4:**
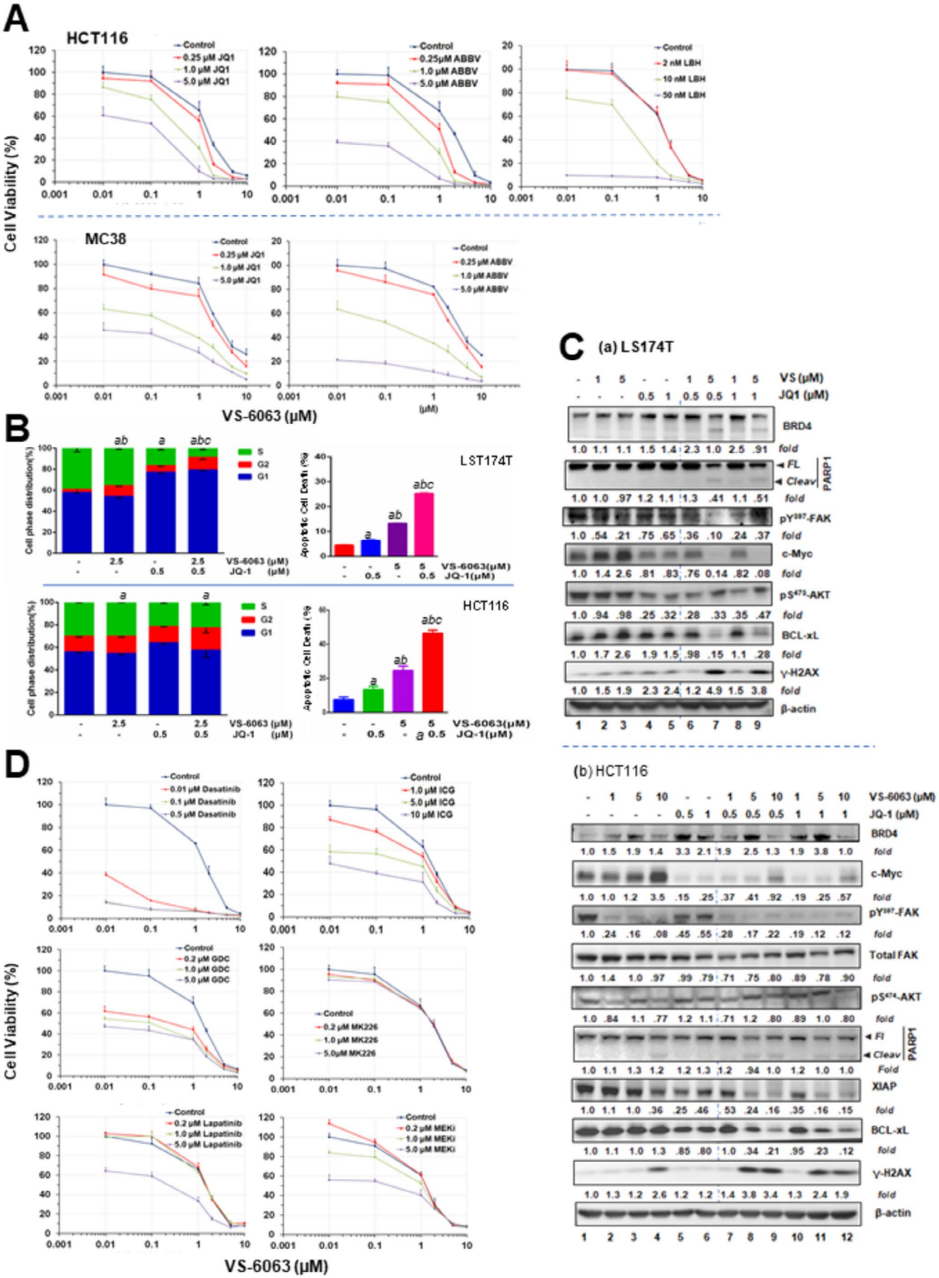
Crosstalk between the integrin–FAK and BRD4–MYC axes in CRC cell growth. Human and mouse CRC cell lines were probed pharmacologically to assess interactions between the integrin–FAK axis and MYC or other oncogenic pathways. (A, D) Cell viability analyses. HCT116 and MC38 cells were treated for 72 h with the indicated doses of various inhibitors, either alone or in combination, followed by MTT assay. Y‐axis: cell viability (mean ± SEM, *n* = 3). (B, C) Cell cycle and apoptosis analyses. CRC cells cultured under 5% FBS were treated with the indicated inhibitors and examined for effects on (B) cell cycle distribution by flow cytometry or (C) apoptosis/biochemical changes by immunoblotting. For cell cycle analysis, triplicate cultures were treated with control (0.1% DMSO) or the indicated inhibitors for 48 h before analysis. Data are shown as mean % (± SEM, *n* = 3) of cells in each phase. Groups with the same letters differ significantly in % G2/M phase or apoptosis (*p* < 0.05, multiple comparisons). For immunoblotting, tumor cells were seeded in 12‐well plates, treated with inhibitors for 24 h, lysed in RIPA buffer, and probed with the indicated antibodies. β‐actin served as loading control. Top panel: LS174T cells treated with VS‐6063 (lanes 2–3, 6–9) and JQ1 (lanes 4–9). Bottom panel: HCT116 cells treated with VS‐6063 (lanes 2–4, 7–12) and JQ1 (lanes 5–12). Lane 1: 0.1% DMSO control.

Based on our recent observations on the intrinsic link between FAK and the BRD4‐MYC axis in solid tumors, we next explored the feasibility of the FAK and BRD4 inhibitors—based co‐inhibition for MYC^high^ CRC tumor cell lines [26, 29]. Our initial profiling analysis showed that only two out of nine cell lines appeared to be sensitive to JQ1, a widely used pan BD domain‐based inhibitor for members of the BET family, primarily BRD4 (Figure S2). However, VS‐6063 exhibited a strong collaboration with either JQ1 or ABBV‐774 in HCT116 or M38 lines, which competitively disrupt the binding of one or two tandem bromodomains (BD1 and BD2) of BRD4 with acetyl groups on histone or transcription factors such as MYC (Figure 4A) [26, 27]. There was also a synergy between VS‐6063 and LBH‐589, an HDAC inhibitor. Meanwhile, the FAK inhibitor (FAKi) and BET inhibitor (BETi) additively or synergistically induced cell cycle arrest at the G1/S and G2/M phases as well as apoptotic cell death in both LS174T and HCT116 lines (Figure 4B). Consistent with these effects, the inhibitor treatment led to a dose‐dependent suppression of MYC, XIAP, Bcl‐xL, and a concomitant increase in the level of cleaved PARP1 and γ‐H2Ax in these cell line models (Figure 4C). While the inhibitor effect on Akt activation (pS^473^) varied between the two lines, the JQ1 treatment consistently restored the level of the long isoform of BRD4 in both cell lines (Figure 4C). Collectively, these analyses unveil a strong synergy or collaboration between integrin‐FAK and BRD4‐MYC axes in CRC in terms of promotion of cell cycle progression and survival.

It is worth noting that we probed a panel of selective chemical inhibitors regarding their abilities to cooperate with VS‐6063 in suppression of cell viability, based on prior studies of oncogenic alteration in the CRC population [2, 3, 4]. The results from these analyses showed that the pharmacological inhibition of RTKs (ErbB2, lapatinib), PI3K (GDC0941), SRC (dasatinib), MEK (U0126) and the Wnt/β‐catenin axis (ICG001), but not Akt (MK226), exhibited a strong collaboration with VS‐6063 in terms of impact on cell viability (Figure 4D). These data are consistent with the output from the bioinformatic analysis of FAK elevation‐associated pathways in the TCGA cohort (Figure 1E).

### 3.4 The Link Between FAK and Metastatic Potential

**FIGURE 5:**
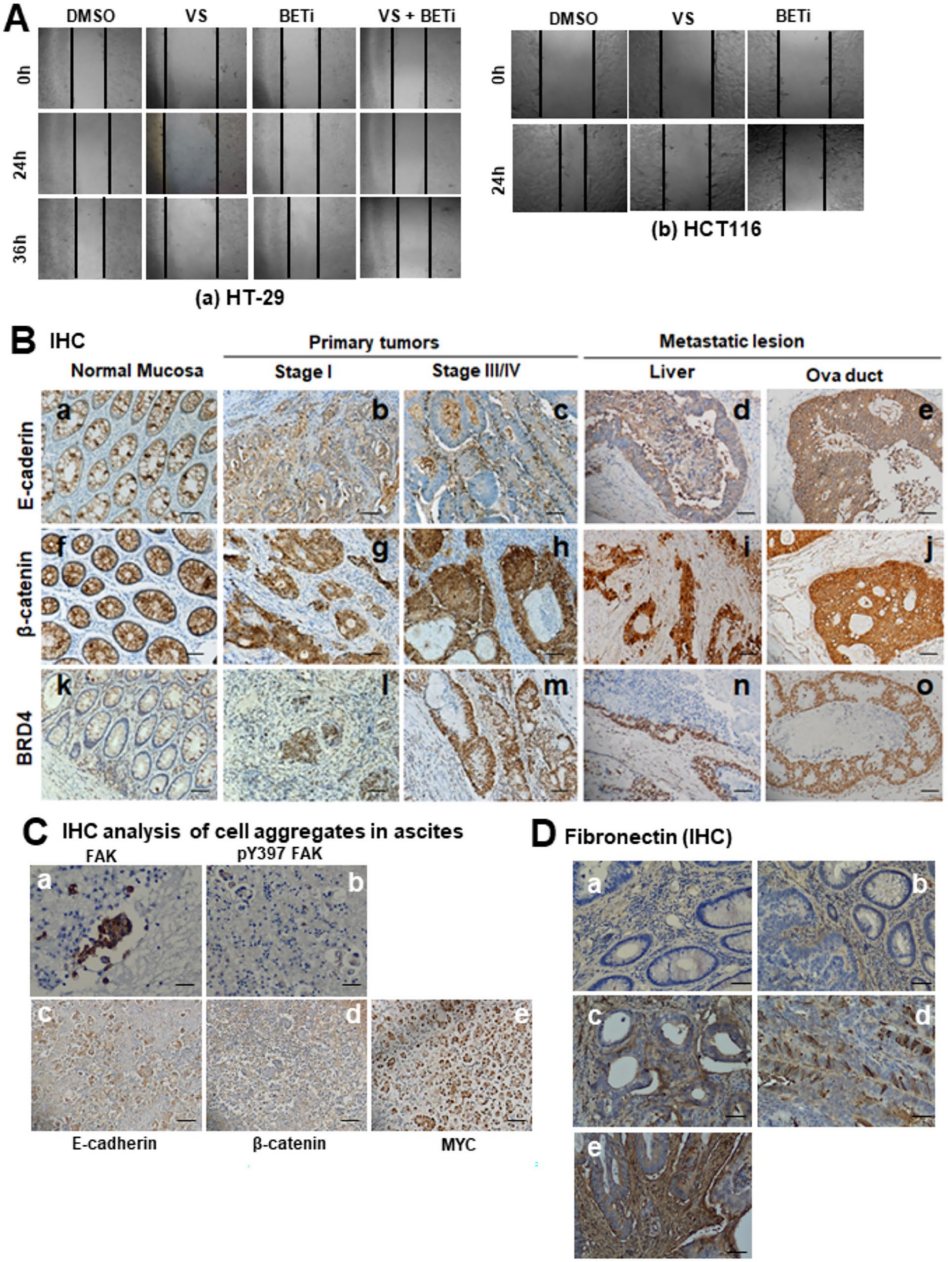
Links between FAK and E‐cadherin, β‐catenin, MYC, BRD4, and YAP1 in pro‐metastatic functions and distant metastasis in vivo. (A) Representative wound‐healing–like migration assays (*n* = 3) showing the effects of FAK inhibition (VS‐6063, “VS”) or BET inhibition (JQ1) on cell motility compared to DMSO control in HT‐29 (a) and HCT116 (b) cells. (B) Representative images of IHC staining of E‐cadherin (a–e), β‐catenin (f–j), and BRD4 (k–o) in normal intestinal mucosa (a, f and k), stage I primary tumors (b, g and l), stage III primary tumors (c, h and m),and distant metastatic lesions in the liver (d, j and n) or oviduct (e, j and o). (C) IHC staining of FAK (a), pY397‐FAK (b), E‐cadherin (c), β‐catenin (d), and MYC (e) in paraffin‐embedded, cell‐enriched pellets derived from patient pleural fluids or ascites. (D) IHC staining of fibronectin in CRC tissues from stage I (a), stage II (b), and stage III (d–e) tumors. Scale bar : 100 μm (all panels).

Both integrin‐FAK axis and MYC oncogene have been implicated in the regulation of pro‐metastatic traits of CRC such as cell motility, EMT, and tumor microenvironments [13, 16, 24, 33, 34]. We tested if they cooperated in the regulation of these traits. As shown in Figure 5A, the FAK inhibitor markedly impaired tumor cell migration in both HT‐29 and HCT116 lines. By contrast, the JQ1 effect was less dramatic. There was also a lack of synergy for FAKi and JQ1. Meanwhile, there was a trend that the percentages of tumors expressing elevated levels of total and active FAK in our local CRC patient cohort increased in the group of metastatic lesions derived from liver, brain, omentum, and ova duct, compared to its staining in stage I tumors (Figure 2, Figure S3, Table S4). Additionally, the expression of MYC, BRD4, β‐catenin, and YAP1 were markedly elevated in metastatic lesions (Figures 2A and 5B). Expression of total FAK and MYC were elevated in patients' ascites (Figure 5C). The portion of tumors with high expression of total FAK correlated with altered expression of MYC and YAP (nuclear) in stage III tumors or metastatic lesions (Tables S5 and S6).

Consistent with the association between FAK and metastatic progression in the CRC cohort, the expression of fibronectin, a ligand for α5β1 integrin and a hallmark for EMT, increased with tumor stage in both stroma and tumors (Figure 5D). Additionally, the treatment with BETi (ABBV‐744 or JQ1) induced an EMT‐like phenotype or cell scattering in Caco2 and HT‐29 lines (Figure S2). This observation is consistent with the role of the long isoform BRD4 in sustaining epithelial cell phenotype described previously [26, 35].

### 3.5 A Role of FAK in Cancer Cell Stemness and Drug Resistance

**FIGURE 6:**
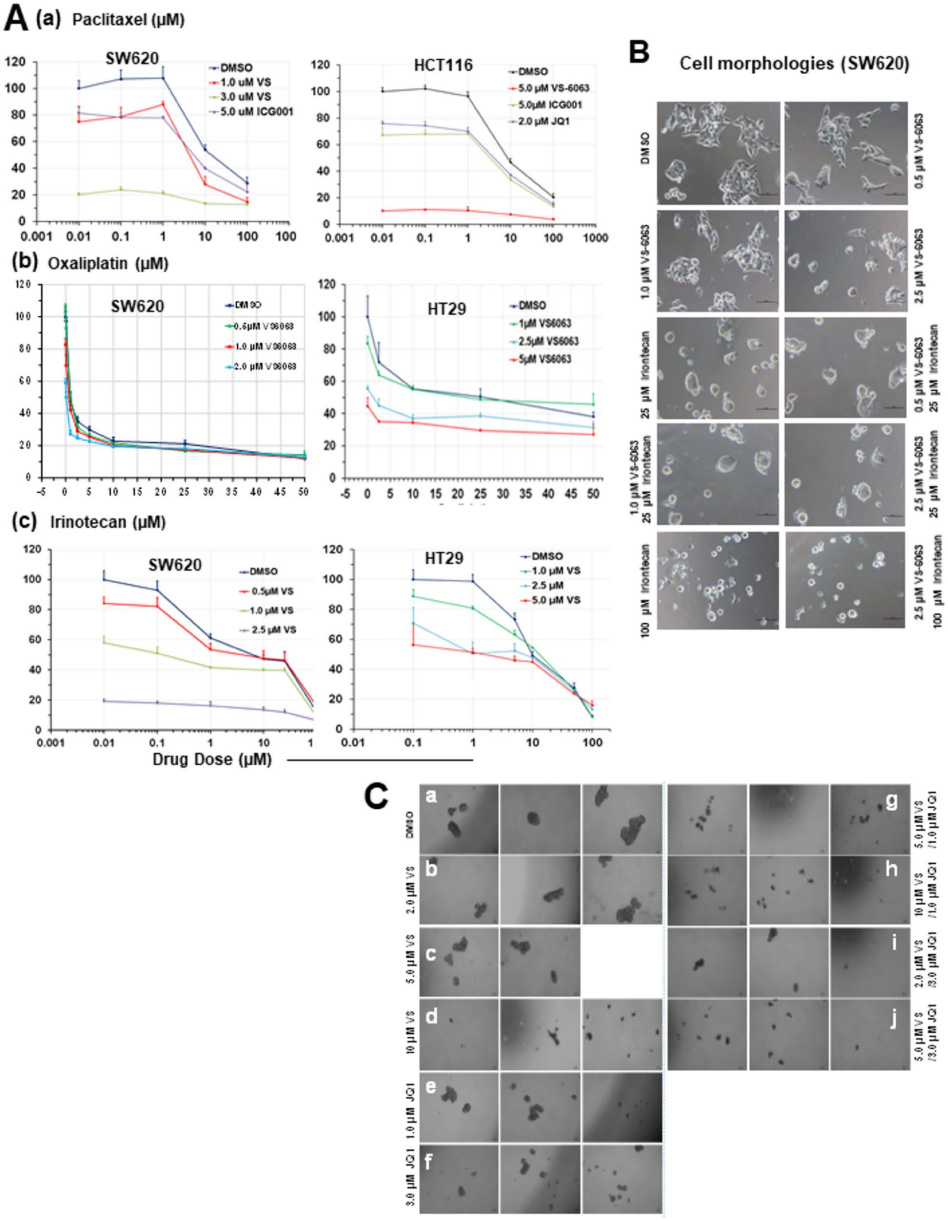
Role of the integrin–FAK signaling axis in CRC stemness and drug sensitivity. (A, B) Effect of FAK inhibition on tumor cell chemosensitivity. (A) Cell viability analysis. CRC cell lines (SW620, HCT116, HT‐29) were seeded in 48‐ or 96‐well plates (triplicates), cultured in 5% FBS overnight, and treated with chemotherapeutic agents (paclitaxel, oxaliplatin, irinotecan) in the presence or absence of FAK inhibitor (VS‐6063, “VS”) for 48–72 h prior to MTT assay. For comparison, the effects of Wnt pathway inhibition (ICG001) or BET inhibition (JQ1) were also tested with paclitaxel and oxaliplatin. X‐axis: drug dose; Y‐axis: cell viability (% of DMSO control, < 0.1% DMSO), mean ± SEM, *n* = 3. (B) Representative images of tumor cells treated with VS‐6063 with or without irinotecan for 24 h. (C) Effect of FAK and BET inhibition on tumorsphere formation (stemness). HCT116 cells were seeded into ultra‐low–attachment 24‐well plates (*n* = 2–3), incubated overnight, and treated with DMSO or the indicated doses of VS‐6063 and JQ1. Tumorspheres were imaged microscopically after 48–60 h. Representative microscopic fields (2–3 per condition) are shown for DMSO and inhibitor treatments (a–i).

We next interrogated if the integrin‐FAK axis played a role in cancer stem cells and related therapeutic resistance in CRC as in other cancer types. As shown in Figure 6A,B, FAK inhibitor treatment promoted tumor cell sensitivity to chemotherapeutic agents such as paclitaxel and oxaliplatin in a dose‐dependent manner, and to a lesser extent, irinotecan. Furthermore, FAKi (VS‐6063) and BETi (JQ1) synergistically impaired tumorsphere formation by HCT116 and LS174T lines (Figure 6C and Figure S4). Collectively, these data suggest a promoting role of FAK and BRD4 in CRC chemoresistance and stemness.

### 3.6 The Clinical Link Between FAK, YAP1, and SRC During CRC Progression

**FIGURE 7:**
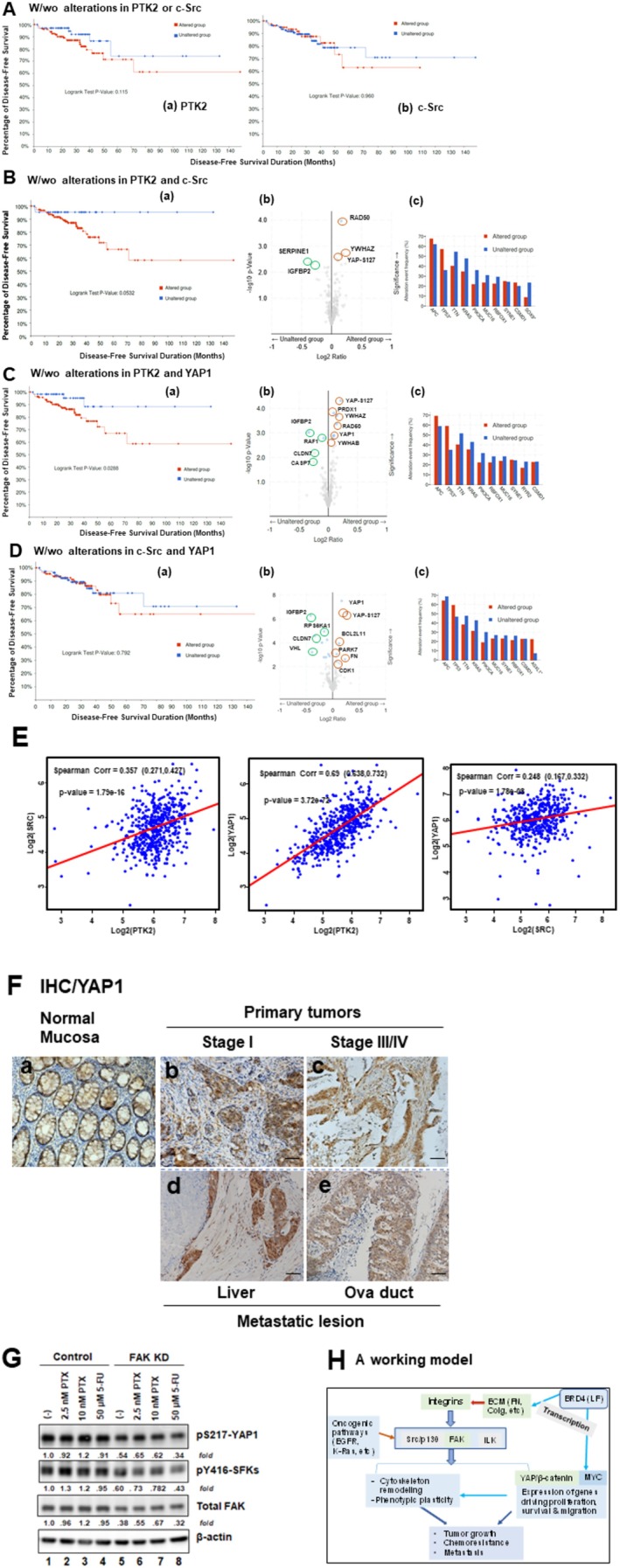
Clinical association of FAK, SRC, and YAP1 with therapeutic relevance in CRC. (A–D) Disease‐free survival (DFS) analysis in the TCGA CRC cohort (PanCancer Atlas, *N* = 594). (a) Kaplan–Meier DFS curves comparing patients with or without genomic alterations, mutations, or dysregulated mRNA expression (*z*‐score ±2 relative to normal; log RNA Seq V2 RSEM) of FAK, SRC, or YAP1. Y‐axis: patient survival (%); X‐axis: DFS time (months). *p* values are indicated. (b) Volcano plots of aberrant protein expression in altered vs unaltered groups. Genes with marked differences are labeled. (c) Profiles of key oncogenic driver gene alterations across groups. (E) Correlation analysis of FAK, c‐Src, and YAP1 expression in the TCGA cohort. (F) Representative IHC staining of YAP1 in normal mucosa (a), stage I tumors (b), stage III/IV tumors (c), and metastatic lesions from liver (d) and oviduct (e). Scale bar: 100 μm. (G) Effect of FAK knockdown ± chemotherapeutic agents on YAP1 and SFK (SRC family kinase) activation. SW620 cells with or without stable FAK knockdown were cultured overnight, treated with paclitaxel (PTX) or 5‐fluorouracil (5‐FU) for 24 h, lysed in RIPA buffer, and immunoblotted. β‐actin served as a loading control. Fold changes are indicated. (H) Proposed working model of functional interactions between the integrin–dependent pathways, YAP1, SRC and the BRD4–MYC axis in CRC progression.

Based on our above screening analysis for the complementary oncogenic pathways of FAK (Figure 4D), we next tested the link between FAK and SRC in terms of disease‐free survival in the TCGA cohort. As shown in Figure 7A,B, the combined dysregulation of FAK and SRC genes was more associated with poor disease‐free survival as compared to the alteration in a single gene. Interestingly, one of the top genes linked to such alteration is the marked change in YAP1 activation, indicated by its pS^127^ phosphorylation. Furthermore, the group of CRC patients with combined dysregulation of YAP1 and FAK, but not SRC, was significantly associated with poor disease‐free survival compared to their counterparts (Figure 7D). However, such difference diminished when the cohort was analyzed under consideration of additional variables such as gender or tumor location (data not shown). Meanwhile, FAK expression appeared more correlated with YAP1 than SRC at the mRNA level (Figure 7E). There was a strong correlation between FAK and YAP1 at the protein level at stages I and III tumor groups in our local CRC patient cohort (Figure 7F, Tables S5–S8). Additionally, FAK knockdown led to a marked decrease in the active form of YAP1 (pS^217^) or c‐Src (pY^416^) in SW620 cells (Figure 7G). Taken together, these observations illustrate a strong link between the FAK and YAP1‐dependent pathway in CRC, particularly regarding disease progression.

## 4 Discussion

The current study has evaluated genomic characteristics, functional roles, clinical significance, and therapeutic potential of the integrin‐FAK axis in two CRC patient populations. Data from the bioinformatic and pathological analyses suggest that FAK is aberrantly expressed and activated in tumor tissues or metastatic lesions. These alterations are associated with the disease progression and are potentiated by SRC and the YAP1‐dependent molecular network. Furthermore, the FAK dependency occurs in a fraction of CRC cell lines probed and is susceptible to the BRD4/MYC axis‐dependent regulation in terms of cell proliferation, survival, EMT, and drug resistance as well as metastatic potential. Overall, the current study has added crucial pathological and molecular evidence on a pro‐tumorigenic and pro‐metastatic role of the integrin‐FAK axis in CRC and boosted its potential as a therapeutic target (Figure 7H).

### 4.1 Clinical Significance of Dysregulation of Integrins and Associated Pathways

While aberrant expression of integrins and their ligands such as collagens and fibronectin have been linked to the CRC malignancy, particularly the CMS2 subtype, its clinical significance and molecular basis remain unclear [5, 36, 37]. Here our analysis shows that many members of the integrin family are aberrantly expressed at the mRNA level in adenocarcinomas, but none of these changes is strongly linked to clinical outcomes or patient survival (data not shown). Instead, FAK, SRC, and YAP1, three key effectors of the integrin‐dependent pathways, are markedly altered at the genomic or transcriptional, posttranslational levels (Figures 1 and 2). The FAK amplification partially overlaps with MYC oncogene and is consistent with their proximity on chromatin 8q24 arm, but the frequency is relatively low compared to other cancer types [26, 27]. Meanwhile, FAK and YAP1 are frequently co‐dysregulated in the CRC patient population and appear associated with disease progression, consistent with their roles in intestinal regeneration, tumor development, and disease recurrence [8, 9, 10, 38, 39].

### 4.2 The Pro‐Proliferative and Pro‐Survival Role of the Integrin‐FAK Axis

The APC disruption‐linked MYC activation is implicated as a crucial driver of FAK expression in CRC [9]. Consistent with this notion, we found that the integrin‐FAK axis promotes cell proliferation in CRC via impacting cell cycle transition to G1/S and G2/M phases and expression of E2F genes, the targets of the MYC oncogene (Figures 1E and 4B). However, FAK may also act upstream of Myc in some of our cell line models examined (Figures 3 and 4). Our detection of a strong collaboration between the integrin‐FAK and BRD4‐MYC axes in the promotion of cell survival is also in line with the crosstalk between the integrin‐FAK axis and the Akt/mTOR or Wnt/β‐catenin pathway in CRC [7, 9, 20, 31], and their links to CRC stemness and disease recurrence [23]. Mechanistically, this functional role may be linked to the activation of the XIAP‐Bcl‐xl pathway (Figure 4), consistent with our analysis of other cancer types [26, 27]. It may also account for the effect of FAK inhibition on tumor cell response to chemotherapeutic agents, particularly paclitaxel and oxaliplatin (Figure 6).

### 4.3 The Pro‐Metastatic Role of the Integrin‐FAK Axis

The integrin‐FAK axis is pro‐metastatic in the context of crosstalk with oncogenic drivers or pathways such as EGFR, c‐MET, and RAS or the TGF‐β pathway [17, 40, 41]. Consistent with this paradigm, the current study found that the percentage of tumors with active FAK (pY^397^ form) increases with tumor stage or distant metastasis in the CRC cohort examined (Figures 2 and 5). Particularly, there was an elevated expression of fibronectin in stroma, a potent ligand for pro‐metastatic α5β1 integrin, implying that FAK may drive metastatic potential through regulation of stromal remodeling, consistent with a recent study [42]. This finding is consistent with a role of the integrin/FAK in the activation of pro‐invasive MMPs [43, 44]. Moreover, our study favors a potential collaboration between FAK and YAP1 in the promotion of tumor metastasis in CRC (Figures 5 and 7). It is in line with the role of YAP1 in sustaining expression of the genes regulating the remodeling of extracellular matrix, such as FOSL, CTGF, and CYR61 [45, 46, 47].

Our study also suggests that the pro‐malignant role of the integrin/FAK axis in CRC is regulated by BRD4. Contrary to the conventional view, BETi treatment seems to induce the EMT‐like phenotype or cell scattering in CRC cells. Conceivably, downregulation of the long isoform BRD4 (Figure 4C) and the integrin‐FAK‐SRC‐YAP1 axis may act in concert to boost expression/activity of EMT‐inducing pro‐metastatic transcription factors such as Snail, as described in a recent study [26].

### 4.4 Molecular Basis of the FAK Activation and Expression of Long‐Form BRD4 Restored by BETi

Despite the promotion of FAK expression by the APC disruption‐linked MYC activation, [9] FAK may be largely activated in CRC through the ECM‐integrin engagement, as the pharmacological inhibitor targeting active FAK (VS‐6063) markedly impairs cell proliferation in several cell lines examined (Figure 3). This notion is also supported by our observation of a strong EMT induction and expression of fibronectin (α5β1 integrin ligand) in cell aggregates in patient ascites or tumor stroma, and an increasing portion of tumors with active FAK with tumor stage or in distant metastasis (Figures 2 and 5). Meanwhile, part of FAK activation in CRC may also stem from dysregulation of tumor suppressor PTEN at the genetic, epigenetic, and posttranscriptional levels in the CRC population [48, 49, 50, 51, 52]. Additionally, FAK in CRC may be activated through its FERM domain engagement with the PIP2 lipid pool on the plasma membrane upon tumor progression [32, 53, 54, 55, 56]. Moreover, epigenetic reader BRD4 may directly bind to the promoter region of integrins or FAK in tumor cells, based on the ChIP‐qPCR analysis in two recent studies [57, 58]. Perhaps, the combinations of integrin activation, aberrant gene transcription/epigenetic regulation, PTEN loss, and reprogramming of membrane lipid composition additively or synergistically drive the hyper‐activation in FAK or integrin‐FAK/YAP1 axis in CRC, ultimately driving tumor growth, metastasis, and drug resistance in CRC.

The dose‐dependent induction of the long isoform BRD4 upon the BETi treatment detected in the current study (Figure 4C) is indicative of the BETi effectiveness, based on a recent clinical study with another BET inhibitor (PLX51107) [59]. Mechanistically, this phenomenon may be linked to the SPOP‐linked proteasomal degradation of BRD4 protein [60]. Alternatively, it may stem from the ER stress‐mediated gene transcription [61]. Aside from a putative suppressor of tumor growth [35, 36], the long‐form BRD4 may impact cellular phenotype through regulation of expression of ECM molecules (e.g., collagen) [62]. At the molecular level, BRD4 has been implicated as an insulator to antagonize DNA damage response through prevention of spreading of histone H2AX phosphorylation on the chromatin. Altogether, this evidence argues for strong molecular and functional roles of BRD4 in CRC and a potential new readout for the BETi‐based targeted therapy.

It is worth noting that there were high molecular‐weight forms of FAK in a fraction of CRC cell line models examined, including SW480, HCT‐8, and Caco2 (Figure 3A). These potential structural mutants may stem from the fusion of FAK and TRAPPC9 genes, according to the genomic profile of FAK in the panel of cancer cell lines described in the Encyclopedia [30, 63] or the CRC cohort in the TCGA database from our cBioPortal‐based analysis (data not shown). They are potentially featured with a structural alteration in the C‐terminal FAT domain of FAK, which may impair the tail‐end‐type auto‐inhibition of FAK activation and lead to its constitutive activation [31].

### 4.5 Therapeutic Potential of Targeting the Integrin‐FAK Axis and Limitation of the Study

Results from the current study strongly support the therapeutic potential of FAK inhibitors as a single agent or as part of combinatorial targeting of other oncogenic drivers such as SRC, BRD4‐MYC axis, and RTKs or chemotherapeutic agents (Figures 1, 3 and 6). In the future evaluation of FAK inhibitor as a single targeting agent, the CRC population may be stratified by MYC, SRC, and YAP1, as there is a lack of strong overlap between FAK activation and the amplification or constitutive activation of SRC and YAP1 in the CRC population (Figure 1). This strategy may also be effective against the metastatic patient cohort, based on our pathological analysis and a recent study [64]. However, FAK‐targeting may encounter adaptive resistance in the clinic, as it exhibits strong crosstalk with RTKs or epigenetic regulators (Figure 4D). Under such a scenario, the co‐targeting of FAK and the BRD4‐MYC axis may be of high therapeutic promise, as there is a functional interdependence between the integrin‐FAK and BRD4‐MYC, a potential “Achilles' heel” for CRC. The state of dysregulated expression or activity of YAP1, SRC, BRD4, PTEN, and MYC may be regarded as putative biomarkers to identify responsive CRC patient population.

The current study also has some limitations. Due to the limited sample size, the evidence on the association between FAP/YAP and patient survival from our analysis of the TCGA cohort (Figure 7A–D) may be weakened after making the Bonferroni correction for additional variables such as sex and tumor location. The interpretation of our data on the aberrant expression of FAK in distant metastasis may need extra caution due to the small pool of metastatic lesions analyzed. Finally, our observation on the suppressive synergy of FAK and BET inhibitors in CRC remains to be verified in vivo with animal or PDX models. Nonetheless, our study has laid out some blueprint for mechanistic understanding of the crosstalk between integrin‐FAK, YAP, BRD4, and MYC in CRC and related therapeutic potential.

## 5 Conclusions

Our current study has demonstrated that the integrin‐FAK pathway in CRC is markedly dysregulated in CRC at the genomic, pathological, and molecular levels. Our functional analysis reveals a strong dependence of a fraction of CRC tumor cell lines on the integrin‐FAK pathway and its crosstalk with YAP1 and the BRD4‐MYC axis. The proliferation or survival of these tumor cell lines is highly susceptible to the inhibition of pharmacological inhibitors of FAK or BET family members. These findings provide a framework for developing therapeutic targeting of the integrin‐FAK pathway alone or in combination with other therapies for CRC treatment.

## Author Contributions

**Rongbo Han:** investigation, visualization, writing – original draft, conceptualization, funding acquisition. **Junfeng Shi:** investigation. **Kai Cheng:** methodology, conceptualization, writing – original draft, investigation, writing – review and editing. **Zian Wang:** investigation, visualization. **Yecang Chen:** investigation, visualization. **Orion Spellecy:** investigation, visualization, project administration. **Abu Saleh Mosa Faisal:** formal analysis, visualization. **Isha Aryal:** investigation. **Jinfei Chen:** conceptualization, supervision. **Rolf Craven:** writing – review and editing. **Olivier Thibault:** writing – review and editing. **Lauren Baldwin:** writing – review and editing. **Lawrence D. Brewer:** writing – review and editing. **Sonia Erfani:** writing – review and editing. **Chi Wang:** formal analysis, supervision. **Zhenheng Guo:** writing – review and editing. **Eric Chen:** writing – review and editing. **Burton Yang:** writing – review and editing. **Frederick Ueland:** writing – review and editing. **Ruihua Guo:** conceptualization, supervision, writing – review and editing. **Xiuwei Yang:** conceptualization, methodology, supervision, funding acquisition, visualization, project administration, resources, writing – original draft, writing – review and editing.

## Ethics Statement

The use of deidentified patients' biospecimens in the study was granted exemption and approved by the institutional IRB ethics committee.

## Conflicts of Interest

The authors declare no conflicts of interest.

## Supporting information


**Figure S1:** Landscape of genomic dysregulation and aberrant mRNA expression of multiple members of integrin family and clinical relevance in the TCGA cohort (PanCancer Atlas, *N* = 526 tumor samples). (A) Genetic mutations, structural variant mutations and mRNA expression were profiled for of genes coding 17 members of integrin family. The cutoff for mRNA Expression with a *z*‐score threshold ±2. Differential expression of proteins was also probed. The threshold of *z*‐score relative to normal samples (log RNA Seq V2 RSEM) was set at ±2. (B) The overall patient survival was plotted for the populations with or without genomic dysregulation for a subset of listed integrin genes. Y axis: Percentage of overall patient survival. X axis: Duration of overall patient survival (months). The threshold of *z*‐score relative to normal samples (log RNA Seq V2 RSEM) was set at ±2. Association between aberrant expression of FAK and patient survival (C).
**Figure S2:** Screening the sensitivity of CRC cell lines to BET inhibitor. (A) Nine CRC cell lines were screened for JQ1 sensitivity via the MTT assay. During the MTT assay, tumor cells were seeded into 96‐well plates o/n, and treated with escalating doses of JQ1 for 48–72 h prior to the assay. Cell viability: calculated % control (0.1% DMSO); mean ± SEM, *n* = 3. The MTT assay was performed with cells cultured under 10% FBS throughout the screening study. (B) Images of Caco2 and HT‐29 cells treated with 0.1% DMSO (control) or inhibitors for 24 h.
**Figure S3:** IHC analysis of expression and distribution of total and active FAK, MYC and β‐catenin in brain‐ and omentum‐derived metastatic lesions. Images of antibody staining of mediators in brain (A–D) and omentum (E–H) metastatic lesions were shown. Scale bar; 50 μm.
**Figure S4:** Analysis of effect of FAKi and BET inhibitors on stemness of CRC cells. The analysis was performed according to the protocol described in Figure 6C. In brief, HCT116 cells were seeded into ultralow 24‐well plates (*n* = 2–3) overnight and followed by treatment with indicated doses of DMSO or VS‐6063 and JQ1, and imaged at 60 h (A–I). Two to three representative microscopic images in a row were shown for each drug treatment (rows A–L).
**Table S1:** Information on the antibodies used in IHC analysis.
**Table S2:** Demographic characteristics of a local CRC patient cohort.
**Table S3:** Major oncogenic features and pathological parameters of a local CRC patient.
**Table S4:** Summary of IHC staining of key mediators across normal intestinal tissues, primary tumors and metastatic lesions.
**Table S5:** Associations of FAK in the stage I tumor group.
**Table S6:** Associations of FAK in the stage II tumor group.
**Table S7:** Associations of pFAK (Y^397^) in the stage II tumor group.
**Table S8:** Associations of FAK in the stage III tumor group.

## Data Availability

The data on genomic and mRNA/protein expression of selected genes in the current study came from the bioinformatic analysis of publicly available TCGA dataset. All infected cell lines with stable gene knockdown are available upon request with MTA.
