# Supplementary material for: Focal Adhesion Kinase Intersects With the BRD4‐MYC Axis and YAP1 to Drive Tumor Cell Growth, Phenotypic Plasticity, Stemness, and Metastatic Potential in Colorectal Cancer
Source: Cancer Med. 2025 Sep 17;14(18):e71227. doi: 10.1002/cam4.71227 (PMC12441738; doi:10.1002/cam4.71227)
Supplement: Supplementary file 1 — Figure S1: Landscape of genomic dysregulation and aberrant mRNA expression of multiple members of integrin family and clinical relevance in the TCGA cohort (PanCancer Atlas, N = 526 tumor samples). (A) Genetic mutations, structural variant mutations and mRNA expression were profiled for of genes coding 17 members of integrin family. The cutoff for mRNA Expression with a z‐score threshold ±2. Differential expression of proteins was also probed. The threshold of z‐score relative to normal samples (log RNA Seq V2 RSEM) was set at ±2. (B) The overall patient survival was plotted for the populations with or without genomic dysregulation for a subset of listed integrin genes. Y axis: Percentage of overall patient survival. X axis: Duration of overall patient survival (months). The threshold of z‐score relative to normal samples (log RNA Seq V2 RSEM) was set at ±2. Association between aberrant expression of FAK and patient survival (C). Figure S2: Screening the sensitivity of CRC cell lines to BET inhibitor. (A) Nine CRC cell lines were screened for JQ1 sensitivity via the MTT assay. During the MTT assay, tumor cells were seeded into 96‐well plates o/n, and treated with escalating doses of JQ1 for 48–72 h prior to the assay. Cell viability: calculated % control (0.1% DMSO); mean ± SEM, n = 3. The MTT assay was performed with cells cultured under 10% FBS throughout the screening study. (B) Images of Caco2 and HT‐29 cells treated with 0.1% DMSO (control) or inhibitors for 24 h. Figure S3: IHC analysis of expression and distribution of total and active FAK, MYC and β‐catenin in brain‐ and omentum‐derived metastatic lesions. Images of antibody staining of mediators in brain (A–D) and omentum (E–H) metastatic lesions were shown. Scale bar; 50 μm. Figure S4: Analysis of effect of FAKi and BET inhibitors on stemness of CRC cells. The analysis was performed according to the protocol described in Figure 6C. In brief, HCT116 cells were seeded into ultralow 24‐well plates (n = 2–3) o [file CAM4-14-e71227-s001.docx]

**Figure S1**. **Landscape of genomic dysregulation and aberrant mRNA expression of multiple members of integrin family and clinical relevance in the TCGA cohort** (PanCancer Atlas, N = 526 tumor samples) ***A*** Genetic mutations, structural variant mutations and mRNA expression were profiled for of genes coding 17 members of integrin family. The cut-off for mRNA Expression with a z-score threshold ± 2. Differential expression of proteins was also probed. The threshold of z-score relative to normal samples (log RNA Seq V2 RSEM) was set at ± 2. ***B*** The overall patient survival was plotted for the populations with or without genomic dysregulation for a subset of listed integrin genes. Y axis: Percentage of overall patient survival. X axis: Duration of overall patient survival (months). The threshold of z-score relative to normal samples (log RNA Seq V2 RSEM) was set at ± 2. Association between aberrant expression of FAK and patient survival (C).

**Figure S2**. **Screening the sensitivity of CRC cell lines to BET inhibitor**. ***A*** Nine CRC cell lines were screened for JQ1 sensitivity via the MTT assay. During the MTT assay, tumor cells were seeded into 96-well plates o/n, and treated with escalating doses of JQ1 for 48-72 hours prior to the assay. Cell viability: calculated % control (0.1% DMSO); mean ± SEM, n =3. The MTT assay was performed with cells cultured under 10% FBS throughout the screening study. ***B*** Images of Caco2 and HT-29 cells treated with 0.1% DMSO (control) or inhibitors for 24 hours.

**Figure S3. IHC analysis of expression and distribution of total and active FAK, MYC and β-catenin in brain- and omentum-derived metastatic lesions**. Images of antibody staining of mediators in brain (a-d) and omentum (e-h) metastatic lesions were shown. Scale bar; 50 µm.

**Figure S4. Analysis of effect of FAKi and BET inhibitors on stemness of CRC cells**. The analysis was performed according to the protocol described in **Fig. 6C**. In brief, HCT116 cells were seeded into ultra-low 24-well plates (n=2-3) overnight and followed by treatment with indicated doses of DMSO or VS-6063 and JQ1, and imaged at 60 hours (a-i). Two to three representative microscopic images in a row were shown for each drug treatment (rows *a*-*l*).

**Table S1. Information on the antibodies used in IHC analysis**

| **Antibody target** | **Dilution** | **Catalogue** | **Clone** | **Vendor** |
| --- | --- | --- | --- | --- |
| Total FAK | 1:200 | sc-558 | C-20 | Santa Cruz Biotechnology |
| pFAK (Y^397^) | 1:100 | Ref:44-624G | polyclonal | Invitrogen |
| β-catenin | RTU* | ZM-0442 | UMAB15 | ZSGB-BIO |
| MYC | 1:100 | ZA-0658 | Y69 | ZSGB-BIO |
| BRD4 | 1:2000 | Ab128874 | EPR5150 (2) | Abcam |
| E-cadherin | RTU | ZA-0565 | EP6 | ZSGB-BIO |
| YAP1 | 1:200 | #4912 | polyclonal | Cell Signaling Technology |

*: Ready-to-use.

**Table S2. Demographic characteristics of a local CRC patient cohort**

|  | **Tissue and tumor types** | | |  | |
| --- | --- | --- | --- | --- | --- |
|  | **Normal (N=26)** | **Primary (N=149)** | **Metastatic (N=18)** | **Total (N=193)** | **P-value** |
| Age |  |  |  |  | 0.5787^1^ |
| N | 26 | 149 | 18 | 193 |  |
| Mean (SD) | 58.3 (12.44) | 59.6 (12.92) | 56.4 (11.17) | 59.1 (12.68) |  |
| Median | 59.0 | 61.0 | 55.0 | 60.0 |  |
| Range | 24.0, 82.0 | 19.0, 83.0 | 32.0, 80.0 | 19.0, 83.0 |  |
|  |  |  |  |  |  |
| Gender, n (%) |  |  |  |  | 0.5641^2^ |
| female | 11 (42.3%) | 56 (37.6%) | 9 (50.0%) | 76 (39.4%) |  |
| male | 15 (57.7%) | 93 (62.4%) | 9 (50.0%) | 117 (60.6%) |  |
|  |  |  |  |  |  |
| Differentiation, n (%) |  |  |  |  | <.0001^2^ |
| high | 0 (0.0%) | 2 (1.4%) | 0 (0.0%) | 2 (1.0%) |  |
| low | 2 (7.7%) | 42 (28.4%) | 18 (100.0%) | 62 (32.3%) |  |
| middle | 24 (92.3%) | 104 (70.3%) | 0 (0.0%) | 128 (66.7%) |  |
|  |  |  |  |  |  |
| Diagnosis, n (%) |  |  |  |  | 0.1377^2^ |
| Non-mucous adenocarcinoma | 21 (80.8%) | 122 (81.9%) | 18 (100.0%) | 161 (83.4%) |  |
| mucous adenocarcinoma | 5 (19.2%) | 27 (18.1%) | 0 (0.0%) | 32 (16.6%) |  |
|  |  |  |  |  |  |
| Tumor size(cm) |  |  |  |  | 0.0052^1^ |
| N | 26 | 131 |  | 157 |  |
| Mean (SD) | 3.6 (1.41) | 4.7 (1.84) |  | 4.5 (1.82) |  |
| Median | 3.0 | 4.2 |  | 4.0 |  |
| Range | 2.0, 6.5 | 1.5, 10.0 |  | 1.5, 10.0 |  |
|  |  |  |  |  |  |
| Neurovascular invasion, n (%) |  |  |  |  | 0.7844^2^ |
| no | 18 (72.0%) | 81 (69.2%) | 0 (.%) | 99 (69.7%) |  |
| yes | 7 (28.0%) | 36 (30.8%) | 0 (.%) | 43 (30.3%) |  |
|  |  |  |  |  |  |
| Lymphatic metastasis, n (%) |  |  |  |  | 0.6359^2^ |
| 0 | 19 (73.1%) | 84 (64.6%) | 0 (.%) | 103 (66.0%) |  |
| 1 | 7 (26.9%) | 39 (30.0%) | 0 (.%) | 46 (29.5%) |  |
| 2 | 0 (0.0%) | 6 (4.6%) | 0 (.%) | 6 (3.8%) |  |
| 3 | 0 (0.0%) | 1 (0.8%) | 0 (.%) | 1 (0.6%) |  |
|  |  |  |  |  |  |
| Tumor stage (TNM), n (%) |  |  |  |  | <.0001^2^ |
| 1 | 8 (30.8%) | 16 (10.7%) | 0 (0.0%) | 24 (12.4%) |  |
| 2 | 11 (42.3%) | 68 (45.6%) | 0 (0.0%) | 79 (40.9%) |  |
| 3 | 7 (26.9%) | 44 (29.5%) | 0 (0.0%) | 51 (26.4%) |  |
| 4 | 0 (0.0%) | 21 (14.1%) | 18 (100.0%) | 39 (20.2%) |  |
| ^1:^ANOVA F-test p-value;  ^2:^ Chi-Square p-value; | | | | | |

**Table S3. Major oncogenic features and pathological parameters of a local CRC patient**

**cohort diagnosed with primary tumors or metastatic disease**

| **Oncogenic alterations**  **/Tumor stage** | **Primary (N=149)** | **Metastatic (N=18)** | **Total (N=193)** | ***p* value^1^** |
| --- | --- | --- | --- | --- |
| K-ras mutation, n (%) |  |  |  | 0.5289 |
| MUT | 56 (52.3%) | 4 (66.7%) | 71 (54.6%) |  |
| WT | 51 (47.7%) | 2 (33.3%) | 59 (45.4%) |  |
|  |  |  |  |  |
| Mismatch repair, n (%) |  |  |  | 0.8408 |
| MSI-H | 8 (7.5%) | 0 (0.0%) | 9 (7.1%) |  |
| MSS | 99 (92.5%) | 4 (100.0%) | 118 (92.9%) |  |
|  |  |  |  |  |
| B-raf mutation, n (%) |  |  |  | 0.6036 |
| B-raf (V600E) | 3 (2.3%) | 0 (.%) | 3 (2.0%) |  |
| wild type | 123 (96.1%) | 0 (.%) | 148 (96.7%) |  |
|  |  |  |  |  |
| Tumor location, n (%) |  |  |  | 0.1095 |
| colon | 89 (59.7%) | 14 (77.8%) | 115 (59.6%) |  |
| rectum | 60 (40.3%) | 4 (22.2%) | 78 (40.4%) |  |
| liver |  |  |  |  |
|  |  |  |  |  |
| pT, n (%) |  |  |  | 0.1719 |
| 1 | 3 (2.2%) | 0 (.%) | 3 (1.8%) |  |
| 2 | 19 (13.8%) | 0 (.%) | 27 (16.5%) |  |
| 3 | 96 (69.6%) | 0 (.%) | 111 (67.7%) |  |
| 4 | 20 (14.5%) | 0 (.%) | 23 (14.0%) |  |
|  |  |  |  |  |
| pN, n (%) |  |  |  | <.0001 |
| 0 | 88 (62.9%) | 0 (0.0%) | 107 (63.7%) |  |
| 1 | 2 (1.4%) | 0 (0.0%) | 2 (1.2%) |  |
| 1a | 18 (12.9%) | 0 (0.0%) | 22 (13.1%) |  |
| 1b | 16 (11.4%) | 0 (0.0%) | 17 (10.1%) |  |
| 2 | 0 (0.0%) | 2 (100.0%) | 2 (1.2%) |  |
| 2a | 11 (7.9%) | 0 (0.0%) | 13 (7.7%) |  |
| 2b | 5 (3.6%) | 0 (0.0%) | 5 (3.0%) |  |
|  |  |  |  |  |
| pM, n (%) |  |  |  | <.0001 |
| 0 | 127 (85.2%) | 0 (0.0%) | 152 (78.8%) |  |
| 1 | 22 (14.8%) | 17 (94.4%) | 40 (20.7%) |  |
| 2 | 0 (0.0%) | 1 (5.6%) | 1 (0.5%) |  |
|  |  |  |  |  |
| Stage(TNM), n (%) |  |  |  | <.0001 |
| 1 | 16 (10.7%) | 0 (0.0%) | 24 (12.4%) |  |
| 2 | 68 (45.6%) | 0 (0.0%) | 79 (40.9%) |  |
| 3 | 44 (29.5%) | 0 (0.0%) | 51 (26.4%) |  |
| 4 | 21 (14.1%) | 18 (100.0%) | 39 (20.2%) |  |

^1:^ Fisher Exact p-value.

**Table S4. Summary of IHC staining of key mediators across normal intestinal tissues, primary tumors and metastatic lesions**

|  | | | | | |
| --- | --- | --- | --- | --- | --- |
|  | Group | | |  | |
|  | Normal (N=26) | Primary (N=149) | Metastatic (N=18) | Total (N=193) | P-value |
| FAK, n (%) |  |  |  |  | <.0001^1^ |
| 0+ | 15 (57.7%) | 15 (10.1%) | 2 (11.1%) | 32 (16.6%) |  |
| 1+ | 11 (42.3%) | 34 (22.8%) | 3 (16.7%) | 48 (24.9%) |  |
| 2+ | 0 (0.0%) | 66 (44.3%) | 8 (44.4%) | 74 (38.3%) |  |
| 3+ | 0 (0.0%) | 34 (22.8%) | 5 (27.8%) | 39 (20.2%) |  |
|  |  |  |  |  |  |
| pFAK, n (%) |  |  |  |  | <.0001^1^ |
| 0+ | 24 (92.3%) | 45 (30.2%) | 2 (18.2%) | 71 (38.2%) |  |
| 1+ | 2 (7.7%) | 70 (47.0%) | 4 (36.4%) | 76 (40.9%) |  |
| 2+ | 0 (0.0%) | 31 (20.8%) | 4 (36.4%) | 35 (18.8%) |  |
| 3+ | 0 (0.0%) | 3 (2.0%) | 1 (9.1%) | 4 (2.2%) |  |
|  |  |  |  |  |  |
| BRD4, n (%) |  |  |  |  | 0.0217^1^ |
| 0+ | 1 (3.8%) | 3 (2.0%) | 0 (0.0%) | 4 (2.2%) |  |
| 1+ | 7 (26.9%) | 20 (13.6%) | 2 (18.2%) | 29 (15.8%) |  |
| 2+ | 15 (57.7%) | 58 (39.5%) | 6 (54.5%) | 79 (42.9%) |  |
| 3+ | 3 (11.5%) | 66 (44.9%) | 3 (27.3%) | 72 (39.1%) |  |
|  |  |  |  |  |  |
| MYC, n (%) |  |  |  |  | <.0001^1^ |
| 0+ | 16 (61.5%) | 28 (18.8%) | 2 (11.1%) | 46 (23.8%) |  |
| 1+ | 10 (38.5%) | 37 (24.8%) | 2 (11.1%) | 49 (25.4%) |  |
| 2+ | 0 (0.0%) | 60 (40.3%) | 7 (38.9%) | 67 (34.7%) |  |
| 3+ | 0 (0.0%) | 24 (16.1%) | 7 (38.9%) | 31 (16.1%) |  |
| Nuclear YAP1, n (%) |  |  |  |  | <.0001^1^ |
| 0+ | 10 (100.0%) | 6 (10.7%) | 2 (11.1%) | 18 (21.4%) |  |
| 1+ | 0 (0.0%) | 11 (19.6%) | 3 (16.7%) | 14 (16.7%) |  |
| 2+ | 0 (0.0%) | 21 (37.5%) | 4 (22.2%) | 25 (29.8%) |  |
| 3+ | 0 (0.0%) | 18 (32.1%) | 9 (50.0%) | 27 (32.1%) |  |
| ^1:^ Fisher Exact p-value. | | | | | |

**Table S5. Associations of FAK in the stage I tumor group**

|  | | | | | | |
| --- | --- | --- | --- | --- | --- | --- |
|  | FAK | | | |  | |
|  | 0+ (N=6) | 1+ (N=9) | 2+ (N=5) | 3+ (N=4) | Total (N=24) | P-value |
| pFAK, n (%) |  |  |  |  |  | 0.0003^1^ |
| 0+ | 6 (100.0%) | 9 (100.0%) | 1 (20.0%) | 1 (25.0%) | 17 (70.8%) |  |
| 1+ | 0 (0.0%) | 0 (0.0%) | 4 (80.0%) | 2 (50.0%) | 6 (25.0%) |  |
| 2+ | 0 (0.0%) | 0 (0.0%) | 0 (0.0%) | 1 (25.0%) | 1 (4.2%) |  |
|  |  |  |  |  |  |  |
| BRD4, n (%) |  |  |  |  |  | 0.5812^1^ |
| 1+ | 3 (50.0%) | 2 (22.2%) | 1 (20.0%) | 0 (0.0%) | 6 (25.0%) |  |
| 2+ | 2 (33.3%) | 4 (44.4%) | 1 (20.0%) | 3 (75.0%) | 10 (41.7%) |  |
| 3+ | 1 (16.7%) | 3 (33.3%) | 3 (60.0%) | 1 (25.0%) | 8 (33.3%) |  |
|  |  |  |  |  |  |  |
| MYC, n (%) |  |  |  |  |  | 0.0738^1^ |
| 0+ | 3 (50.0%) | 4 (44.4%) | 0 (0.0%) | 0 (0.0%) | 7 (29.2%) |  |
| 1+ | 2 (33.3%) | 0 (0.0%) | 1 (20.0%) | 0 (0.0%) | 3 (12.5%) |  |
| 2+ | 1 (16.7%) | 5 (55.6%) | 3 (60.0%) | 3 (75.0%) | 12 (50.0%) |  |
| 3+ | 0 (0.0%) | 0 (0.0%) | 1 (20.0%) | 1 (25.0%) | 2 (8.3%) |  |
|  |  |  |  |  |  |  |
| Nuclear YAP1, n (%) |  |  |  |  |  | 0.0361^1^ |
| 0+ | 4 (100.0%) | 4 (66.7%) | 0 (0.0%) | 0 (0.0%) | 8 (61.5%) |  |
| 1+ | 0 (0.0%) | 1 (16.7%) | 0 (0.0%) | 1 (100.0%) | 2 (15.4%) |  |
| 2-3+ | 0 (0.0%) | 1 (16.7%) | 2 (100.0%) | 0 (0.0%) | 3 (23.1%) |  |
| ^1:^ Fisher Exact p-value. | | | | | | |

**Table S6. Associations of FAK in the stage II tumor group**

|  | | | | | | |
| --- | --- | --- | --- | --- | --- | --- |
|  | FAK | | | |  | |
|  | 0+ (N=15) | 1+ (N=20) | 2+ (N=27) | 3+ (N=17) | Total (N=79) | P-value |
| **pFAK**, n (%) |  |  |  |  |  | <.0001^1^ |
| 0+ | 14 (93.3%) | 12 (60.0%) | 8 (29.6%) | 0 (0.0%) | 34 (43.0%) |  |
| 1+ | 1 (6.7%) | 8 (40.0%) | 18 (66.7%) | 9 (52.9%) | 36 (45.6%) |  |
| 2+ | 0 (0.0%) | 0 (0.0%) | 1 (3.7%) | 8 (47.1%) | 9 (11.4%) |  |
|  |  |  |  |  |  |  |
| **BRD4**, n (%) |  |  |  |  |  | 0.0661^1^ |
| 0+ | 3 (20.0%) | 0 (0.0%) | 0 (0.0%) | 0 (0.0%) | 3 (3.8%) |  |
| 1+ | 3 (20.0%) | 4 (20.0%) | 2 (7.4%) | 1 (5.9%) | 10 (12.7%) |  |
| 2+ | 6 (40.0%) | 10 (50.0%) | 10 (37.0%) | 7 (41.2%) | 33 (41.8%) |  |
| 3+ | 3 (20.0%) | 6 (30.0%) | 15 (55.6%) | 9 (52.9%) | 33 (41.8%) |  |
|  |  |  |  |  |  |  |
| MYC, n (%) |  |  |  |  |  | 0.0759^1^ |
| 0+ | 8 (53.3%) | 3 (15.0%) | 6 (22.2%) | 1 (5.9%) | 18 (22.8%) |  |
| 1+ | 4 (26.7%) | 9 (45.0%) | 6 (22.2%) | 5 (29.4%) | 24 (30.4%) |  |
| 2+ | 2 (13.3%) | 6 (30.0%) | 10 (37.0%) | 5 (29.4%) | 23 (29.1%) |  |
| 3+ | 1 (6.7%) | 2 (10.0%) | 5 (18.5%) | 6 (35.3%) | 14 (17.7%) |  |
|  |  |  |  |  |  |  |
| Nuclear β-catenin,  n (%) |  |  |  |  |  | 0.0452^1^ |
| 0+ | 14 (93.3%) | 13 (65.0%) | 14 (51.9%) | 12 (70.6%) | 53 (67.1%) |  |
| 3+ | 1 (6.7%) | 7 (35.0%) | 13 (48.1%) | 5 (29.4%) | 26 (32.9%) |  |
|  |  |  |  |  |  |  |
| Nuclear YAP1, n (%) |  |  |  |  |  | 0.1516^1^ |
| 0+ | 1 (50.0%) | 2 (40.0%) | 3 (27.3%) | 0 (0.0%) | 6 (26.1%) |  |
| 1+ | 1 (50.0%) | 1 (20.0%) | 1 (9.1%) | 4 (80.0%) | 7 (30.4%) |  |
| 2+ | 0 (0.0%) | 1 (20.0%) | 6 (54.5%) | 1 (20.0%) | 8 (34.8%) |  |
| 3+ | 0 (0.0%) | 1 (20.0%) | 1 (9.1%) | 0 (0.0%) | 2 (8.7%) |  |
| Missing | 13 | 15 | 16 | 12 | 56 |  |
| ^1:^ Fisher Exact p-value. | | | | | | |

**Table S7. Associations of pFAK (Y^397^) in the stage II tumor group**

|  | **pFAK** | | |  | |
| --- | --- | --- | --- | --- | --- |
|  | 0+ (N=34) | 1+ (N=36) | 2+ (N=9) | Total (N=79) | P-value |
| BRD4, n (%) |  |  |  |  | 0.4040^1^ |
| 0+ | 3 (8.8%) | 0 (0.0%) | 0 (0.0%) | 3 (3.8%) |  |
| 1+ | 6 (17.6%) | 3 (8.3%) | 1 (11.1%) | 10 (12.7%) |  |
| 2+ | 14 (41.2%) | 16 (44.4%) | 3 (33.3%) | 33 (41.8%) |  |
| 3+ | 11 (32.4%) | 17 (47.2%) | 5 (55.6%) | 33 (41.8%) |  |
|  |  |  |  |  |  |
| MYC, n (%) |  |  |  |  | 0.0025^1^ |
| 0+ | 13 (38.2%) | 5 (13.9%) | 0 (0.0%) | 18 (22.8%) |  |
| 1+ | 14 (41.2%) | 8 (22.2%) | 2 (22.2%) | 24 (30.4%) |  |
| 2+ | 5 (14.7%) | 14 (38.9%) | 4 (44.4%) | 23 (29.1%) |  |
| 3+ | 2 (5.9%) | 9 (25.0%) | 3 (33.3%) | 14 (17.7%) |  |
|  |  |  |  |  |  |
| Nuclear β-catenin, n (%) |  |  |  |  | 0.5193^1^ |
| 0+ | 25 (73.5%) | 22 (61.1%) | 6 (66.7%) | 53 (67.1%) |  |
| 3+ | 9 (26.5%) | 14 (38.9%) | 3 (33.3%) | 26 (32.9%) |  |
|  |  |  |  |  |  |
| Nuclear YAP1, n (%) |  |  |  |  | 0.5659^1^ |
| 0+ | 3 (33.3%) | 3 (25.0%) | 0 (0.0%) | 6 (26.1%) |  |
| 1+ | 1 (11.1%) | 4 (33.3%) | 2 (100.0%) | 7 (30.4%) |  |
| 2+ | 4 (44.4%) | 4 (33.3%) | 0 (0.0%) | 8 (34.8%) |  |
| 3+ | 1 (11.1%) | 1 (8.3%) | 0 (0.0%) | 2 (8.7%) |  |
| ^1:^ Fisher Exact p-value. | | | | | |

**Table S8. Associations of FAK in**  **the stage III tumor group**

|  | **Total FAK** | | | |  | |
| --- | --- | --- | --- | --- | --- | --- |
|  | 0+ (N=8) | 1+ (N=12) | 2+ (N=20) | 3+ (N=11) | Total (N=51) | P-value |
| pFAK, n (%) |  |  |  |  |  | <.0001^1^ |
| 0+ | 8 (100.0%) | 7 (58.3%) | 2 (10.0%) | 0 (0.0%) | 17 (33.3%) |  |
| 1+ | 0 (0.0%) | 4 (33.3%) | 13 (65.0%) | 3 (27.3%) | 20 (39.2%) |  |
| 2+ | 0 (0.0%) | 1 (8.3%) | 5 (25.0%) | 7 (63.6%) | 13 (25.5%) |  |
| 3+ | 0 (0.0%) | 0 (0.0%) | 0 (0.0%) | 1 (9.1%) | 1 (2.0%) |  |
|  |  |  |  |  |  |  |
| BRD4, n (%) |  |  |  |  |  | 0.7471^1^ |
| 0+ | 1 (12.5%) | 0 (0.0%) | 0 (0.0%) | 0 (0.0%) | 1 (2.0%) |  |
| 1+ | 1 (12.5%) | 4 (33.3%) | 3 (15.0%) | 2 (18.2%) | 10 (19.6%) |  |
| 2+ | 4 (50.0%) | 5 (41.7%) | 9 (45.0%) | 4 (36.4%) | 22 (43.1%) |  |
| 3+ | 2 (25.0%) | 3 (25.0%) | 8 (40.0%) | 5 (45.5%) | 18 (35.3%) |  |
|  |  |  |  |  |  |  |
| MYC, n (%) |  |  |  |  |  | 0.0197^1^ |
| 0+ | 6 (75.0%) | 1 (8.3%) | 5 (25.0%) | 1 (9.1%) | 13 (25.5%) |  |
| 1+ | 2 (25.0%) | 6 (50.0%) | 5 (25.0%) | 2 (18.2%) | 15 (29.4%) |  |
| 2+ | 0 (0.0%) | 2 (16.7%) | 8 (40.0%) | 6 (54.5%) | 16 (31.4%) |  |
| 3+ | 0 (0.0%) | 3 (25.0%) | 2 (10.0%) | 2 (18.2%) | 7 (13.7%) |  |
|  |  |  |  |  |  |  |
| Nuclear YAP1, n (%) |  |  |  |  |  | 0.0238^1^ |
| 0+ | 0 (.%) | 2 (50.0%) | 0 (0.0%) | 0 (0.0%) | 2 (16.7%) |  |
| 1+ | 0 (.%) | 1 (25.0%) | 2 (33.3%) | 0 (0.0%) | 3 (25.0%) |  |
| 2+ | 0 (.%) | 1 (25.0%) | 4 (66.7%) | 0 (0.0%) | 5 (41.7%) |  |
| 3+ | 0 (.%) | 0 (0.0%) | 0 (0.0%) | 2 (100.0%) | 2 (16.7%) |  |
| ^1:^ Fisher Exact p-value. | | | | | | |
